# Combined TLR7/8 and TLR9 Ligands Potentiate the Activity of a *Schistosoma japonicum* DNA Vaccine

**DOI:** 10.1371/journal.pntd.0002164

**Published:** 2013-04-04

**Authors:** Xuefeng Wang, Liyang Dong, Hongchang Ni, Sha Zhou, Zhipeng Xu, Jason Shih Hoellwarth, Xiaojun Chen, Rongbo Zhang, Qiaoyun Chen, Feng Liu, Jun Wang, Chuan Su

**Affiliations:** 1 Department of Central Laboratory, The Affiliated People's Hospital, Jiangsu University, Zhenjiang, Jiangsu, People's Republic of China; 2 Department of Pathogen Biology and Immunology, School of Medicine, Anhui University of Science and Technology, Huainan, Anhui, People's Republic of China; 3 Department of Pathogen Biology and Immunology, Jiangsu Key Laboratory of Pathogen Biology, Nanjing Medical University, Nanjing, Jiangsu, People's Republic of China; 4 Department of General Surgery, Keck School of Medicine of the University of Southern California, Los Angeles, California, United States of America; University of Queensland, Australia

## Abstract

**Background:**

Toll-like receptor (TLR) ligands have been explored as vaccine adjuvants for tumor and virus immunotherapy, but few TLR ligands affecting schistosoma vaccines have been characterized. Previously, we developed a partially protective DNA vaccine encoding the 26-kDa glutathione S-transferase of *Schistosoma japonicum* (pVAX1-Sj26GST).

**Methodology/Principal Findings:**

In this study, we evaluated a TLR7/8 ligand (R848) and a TLR9 ligand (CpG oligodeoxynucleotides, or CpG) as adjuvants for pVAX1-Sj26GST and assessed their effects on the immune system and protection against *S. japonicum*. We show that combining CpG and R848 with pVAX1-Sj26GST immunization significantly increases splenocyte proliferation and IgG and IgG2a levels, decreases CD4^+^CD25^+^Foxp3^+^ regulatory T cells (Treg) frequency *in vivo*, and enhances protection against *S. japonicum*. CpG and R848 inhibited Treg-mediated immunosuppression, upregulated the production of interferon (IFN)-γ, tumor necrosis factor (TNF)-α, interleukin (IL)-4, IL-10, IL-2, and IL-6, and decreased Foxp3 expression *in vitro*, which may contribute to prevent Treg suppression and conversion during vaccination and allow expansion of antigen-specific T cells against pathogens.

**Conclusions:**

Our data shows that selective TLR ligands can increase the protective efficacy of DNA vaccines against schistosomiasis, potentially through combined antagonism of Treg-mediated immunosuppression and conversion.

## Author Summary

There is evidence that TLR activation can block Treg cell responses and thereby break tolerance to self-antigens. It is expected that the use of TLR ligands as vaccine adjuvants will induce potent anti-pathogen immune responses and simultaneously overcome immune inhibition mediated by Tregs. However, the impact of TLR ligands on schistosomiasis vaccines is unclear. Here, we demonstrate that the use of a TLR7/8 ligand (R848) and a TLR9 ligand (CpG) as adjuvants in combination with the *S. japonicum* vaccine pVAX1-Sj26GST improves disease protection. The combination of CpG and R848 administered after vaccination causes an immune response marked by an upregulation of splenocyte proliferation and IgG and IgG2a levels that also coincides with a decreased proportion of CD4^+^CD25^+^ Tregs in mice. We also show that combined adjuvant use of CpG and R848 may impair Treg development and function by promoting the secretion of proinflammatory cytokines and reducing Foxp3 expression. Our findings suggest that in combination with the vaccine, TLR ligands may protect the effector response from Treg-mediated suppression, thereby eliciting the appropriate immune response to improve vaccine efficacy. Immunization combined with the TLR ligands CpG and R848 thus represents a promising new approach for the design of schistosoma vaccines.

## Introduction

Schistosomiasis is regarded as one of the most neglected tropical diseases (NTDs) of high importance, and remains a major problem in public health in endemic countries [1], [2]. Although schistosomiasis can be treated with the drug praziquantel [3], high reinfection rates limit its overall success, where repeated administering is often necessary multiple times during the first two decades [4], [5]. Therefore, the development of a safe and effective vaccine would improve the long-term treatment of schistosomiasis and should improve the efficacy of therapeutic interventions [6], [7]. Despite decades of effort developing vaccines against schistosoma, including *Schistosoma japonicum (S. japonicum)*, the current schistosoma vaccine induces only limited protection for reasons that remain unclear.

A potential issue limiting the immune response to vaccination is the presence of regulatory T cells (Tregs) that suppress T cell activation [8], [9]. Multiple studies in mice have shown that Tregs dampen the immune response against pathogens, including *S. japonicum* [10], [11]. Increased levels of Tregs have been documented in the peripheral blood of schistosoma-infected patients [12]. Furthermore, naturally occurring CD4^+^CD25^+^ Tregs as well as adaptive CD25^+^Foxp3^+^ Tregs, Tr1 cells, and Th3 cells have all been detected in schistosoma-infected mice [13], [14]. Treg depletion improves the efficacy of vaccines against pathogens in mice [15], [16]. Therefore, vaccine strategies that target both the innate and adaptive immune systems for the generation/upregulation of potent anti-pathogen immune responses and simultaneously overcome Treg-mediated immune inhibition are more likely to succeed.

Toll-like receptors (TLRs) are mediators of innate immune responses that detect conserved pathogen-associated molecules. Binding of TLRs with their specific ligands induces a signaling cascade resulting in the induction of type I IFNs and other cytokines, which drive an inflammatory response and activate the adaptive immune systems [17]. As TLRs provide an important link between innate and adaptive immunity, TLR ligands are increasingly being used in the development of pathogen vaccines [18].

In addition to activating effector T cells (Teffs), TLR agonists can indirectly or directly modulate the function of Treg cells. There is evidence that TLR activation can block Treg cellular responses, thereby breaking tolerance to self-antigens. For instance, the TLR9 ligand CpG can synergize with anti-CD3 to partially abrogate the suppressive activity of Tregs [19], [20]. Synthetic and natural ligands for human TLR8 can also reverse Tregs function independently of dendritic cells (DCs) [21]. Given that certain TLR ligands, in particular the TLR7/8 ligand resiquimod (R848), and the TLR9 ligand CpG, can elicit a strong immune response, these ligands may be used as adjuvants during treatment of virus-infected or cancer patients with high numbers of Tregs [22], [23]. To date, few TLR ligands affecting schistosoma vaccines have been characterized [24].

We recently developed a partially protective DNA vaccine encoding the 26-kDa glutathione S-transferase of *S. japonicum* (pVAX1-Sj26GST) and demonstrated that an abundance of CD4^+^CD25^+^ Tregs induced after pVAX1-Sj26GST vaccination may explain the limited protection conferred by this vaccine [25]. Given that R848 and CpG can elicit strong immune responses and potentially inhibit the suppression of Tregs during vaccination [23], [26], we tested whether combining CpG and/or R848 with the pVAX1-Sj26GST vaccine would obtain better immune efficacy against *S. japomicum* and assessed the impact of these TLR ligands on Treg function *in vitro*. Following pVAX1-Sj26GST vaccination, we found that combined use CpG of R848 significantly enhances splenocyte proliferation and IgG and IgG2a levels, increases IFN-γ and TNF-α levels in the supernatant of splenocytes, and improves immune protection against *S. japonicum*. The combination of CpG and R848 inhibited Treg-mediated immunosuppression, upregulated the production of IFN-γ, TNF-α, IL-4, IL-10, IL-2, and IL-6, and decreased Foxp3 expression *in vitro*, which collectively may contribute to prevent Treg suppression and conversion during vaccination and allow expansion of schistosome antigen-specific T cells. The combination of an *S. japonicum* DNA vaccine with the TLR ligands CpG and R848 thus represents a promising new approach for the design and implementation of schistosome vaccination.

## Materials and Methods

### Ethics statement

Animal experiments were performed in strict accordance with the Regulations for the Administration of Affairs Concerning Experimental Animals (1988.11.1), and all efforts were made to minimize suffering. All animal procedures were approved by the Institutional Animal Care and Use Committee (IACUC) of Nanjing Medical University for the use of laboratory animals (Permit Number: NJMU 09-1107).

### Animal studies

Six-week-old C57BL/6 female mice were provided by the Center of Experimental Animals (Nanjing University, Nanjing, China) and bred in university facilities. The experimental protocol was approved by the Institutional Animal Care and Use Committee (IACUC) as previously described [25]. *Oncomelania hupensis* harboring *S. japonicum* cercariae (Chinese mainland snail strain) were purchased from the Jiangsu Institute of Parasitic Diseases (Wuxi, China).

### TLR ligands and antigen preparation

The TLR9 ligand CpG oligodeoxynucleotides (ODN) 1826 (CpG; 5′-TCCATGACGTTCCTGACGTT-3′), with a nuclease-resistant phosphorothioate backbone and no detectable endotoxin, was purchased from Coley Pharmaceutical Group (Wellesley, MA, USA).

The TLR7/8 ligand resiquimod (R848) was purchased from Invivogen (Toulouse, France). Soluble schistosome worm antigen (SWA) was prepared as previously described [25], [27].

### DNA vaccine preparation

The construction, expression, and purification of pVAX1-Sj26GST and pVAX1 have previously been described [25], [28]. Sj26GST sequence: GenBank accession no. M14654.1 (http://www.ncbi.nlm.nih.gov/nuccore/160926); UniProtKB/Swiss-Prot accession no. P08515.3 (http://www.ncbi.nlm.nih.gov/protein/P08515.3). For *in vivo* experiments, all plasmids were prepared using the Qiagen Endo-Free Plasmid Kit (Qiagen, Valencia, CA). The Limulus Amebocyte Lysate QCL-1000 Kit (Cambrex, Charles City, IA, USA) was used to confirm that the endotoxin concentrations were below 0.1 endotoxin units (EU) per dose.

### Immunization and challenge infection

For the characterization of immune responses, three independent experiments were performed. In each experiment, C57BL/6 mice (6 mice per group) were injected with pVAX1-Sj26GST (50 µg), with or without 25 µg CpG, 25 µg R848, or both CpG (25 µg) and R848 (25 µg). The DNA vaccine was delivered intramuscularly into the left tibialis anterior muscle in a total volume of 50 µL PBS. CpG and/or R848 were injected subcutaneously in 100 µL PBS at the base of the tail. As negative controls, mice were treated with pVAX1, R848, or CpG only. The immunization was repeated 3 times at 14-day intervals. One week after the final vaccination, mice were sacrificed for characterization of cellular and humoral immune responses. CpG and R848 dosing and subcutaneous injection method were based on previous publications [29], [30].

For the vaccination challenge trial, 2 independent experiments were carried out. In each experiment, C57BL/6 mice were divided into 7 groups of 8 mice each. Each mouse was injected with pVAX1-Sj26GST (50 µg), with or without CpG (25 µg), R848 (25 µg), or both CpG (25 µg) and R848 (25 µg) as above. Immunization was repeated 3 times at 14-day intervals. Two weeks after the final vaccination, all mice from each group were challenged percutaneously with 40±1 *S. japonicum* cercariae. After 6 weeks, mice were sacrificed and perfused to determine the adult worm burdens and liver egg burdens. Reductions in worms/liver egg burdens are expressed as a percentage of the burden recorded in the pVAX1 control group.

### Antibody detection in the sera of immunized mice

For antibody detection, serum samples were collected 7 days after the last immunization. Standard ELISAs were performed using SWA as the antigen source, which was prepared as previously described [25], [27], [28]. Antibody detection in the sera of immunized mice was performed as previously described [25], [31]. In brief, ELISA plates (Titertek Immuno Assay-Plate, ICN Biomedicals Inc., Costa Mesa, CA, USA) were coated with SWA (15 µg/mL) in 50 mM carbonate buffer (pH 9.6) and stored overnight at 4°C. Plates were washed and developed using tetramethylbenzidine (TMB) substrate (BD Biosciences Pharmigen, San Diego, CA). The enzymatic reaction was stopped with 1N H_2_SO_4_ and plates were read at a 450-nm wavelength. To analyze IgG, IgG1, IgG2a, and IgM, mouse-specific secondary antibodies (Bio-Rad, Hercules, CA, USA) were used at a dilution of 1∶1000. All samples were assayed in triplicate.

To determine the titers of antibodies after the last immunization, the sera from mouse within a group were pooled, serially diluted, and analyzed by ELISA as described above. All samples were assayed in triplicate. End-point titers were defined as the highest plasma dilution that resulted in an absorbance value (OD 450 nm) two times greater than that of non-immune plasma with a cut-off value of 0.05.

### Splenocyte proliferation response and cytokine determination

[^3^H] thymidine (^3^H-TdR) incorporation was used to measure splenocyte proliferation. Seven days after the last immunization, 6 mice from each group were sacrificed and splenocytes were harvested. In 96-well plates, 2×10^5^ cells per well were incubated for 72 h in 200 µL of complete media in the presence of SWA (15 µg/mL). After 56 h in culture, 0.5 µCi [^3^H] thymidine (Amersham, Burkinghamshire, UK) was added to each well. At the end of the incubation period, the cells were harvested on filters and the incorporated [^3^H] thymidine was counted.

To evaluate cytokine production, single-cell suspensions of splenocytes were cultured in the presence of 15 µg/mL SWA or control medium at 2×10^5^ cells/well in round bottom 96-well plates. After 3 days, culture supernatants were collected and assayed for IFN-γ, TNF-α, IL-4, and IL-10 using the FlowCytomix Mouse Cytokine Kit (Bender MedSystems, Vienna, Austria) according to the manufacturer's instructions.

### Cell isolation

Single cell suspensions were prepared by teasing apart spleens and inguinal and mesenteric lymph nodes (LNs) from 6 mice per group in PBS containing 1% FCS and 1% EDTA followed by red blood cell (RBC) lysis with Tris ammonium chloride buffer. CD4^+^ T cells were purified from single cell suspensions with a CD4^+^ T cell negative-isolation kit (Miltenyi Biotec, Auburn, CA) and a magnetic activated cell sorter (MACS) according to the manufacturer's recommendations (>97% CD4^+^ T cells by flow cytometric analysis).

CD4^+^CD25^+^ and CD4^+^CD25^−^ cell populations were separated from purified CD4^+^ T cells using a mouse regulatory T cell isolation kit (Miltenyi Biotec) following the manufacturer's protocol. The CD25^+^ populations were >95% CD4^+^CD25^+^, and the CD4^+^CD25^−^ populations were 98% pure as determined by flow cytometry.

Antigen presenting cells (APCs) were obtained from single cell suspensions by depleting T cells using a mixture of magnetic beads conjugated with either anti-CD8 or anti-CD4 monoclonal antibodies (mAb) (Miltenyi Biotec) followed by irradiation (30 Gy).

### Cell culture

For suppression assays, 1×10^5^ CD4^+^CD25^−^ T cells/well, 5×10^4^ CD4^+^CD25^+^ T cells/well or both were cultured in 96-well U-bottom plates with 1×10^5^ APCs/well in triplicate for 72 h at 37°C in complete RPMI 1640 medium (0.2 mL/well). Cultures were stimulated with 1 µg/mL soluble anti-CD3 (BD Pharmingen, San Diego, CA) in the presence or absence of 3 µg/mL CpG, 3 µg/mL R848, or a combination of both (3 µg/mL each of CpG and R848). Proliferation was measured by incubating cells with 0.5 µCi/well ^3^H thymidine and measuring ^3^H thymidine incorporation during the final 16 h of a 3-d culturing period. The supernatants were collected to quantitatively measure IFN-γ, TNF-α, IL-4, IL-10, IL-2, and IL-6 production using the FlowCytomix Mouse Cytokine Kit (Bender MedSystems, Vienna, Austria) according to the manufacturer's instructions.

### Flow cytometry

For analysis of CD4^+^CD25^+^Foxp3^+^ T cells, the Mouse Regulatory T Cell Staining Kit (eBioscience, San Diego, CA) was used. Splenocytes from immunized mice or naïve mice incubated in the presence or absence of CpG (3 µg/mL), R848 (3 µg/mL), or a combination of both (3 µg/mL each of CpG and R848) for 48 h, were surface-stained with PerCP anti-CD3 monoclonal antibody (mAb); (eBioscience, San Diego, CA), FITC–anti-CD4 mAb, and APC–anti-CD25 mAb, followed by fixation and permeabilization with Cytofix/Cytoperm and intracellular staining with phycoerythrin (PE)-mouse anti-Foxp3 or PE–IgG2a rat IgG control antibody according to the manufacturer's protocol. Data were collected on a FACSCalibur flow cytometer using CellQuest (BD Biosciences, Franklin Lakes, NJ) and analyzed with FlowJo software (Tree Star, San Carlos, CA).

### Statistical analysis

Statistical analyses were performed using SPSS version 10.1 (Statistical Package for Social Sciences statistical software, Chicago, IL). Results were expressed as the mean ± standard error of the mean (SEM). The Mann-Whitney *U* test was used to calculate the significance between the different groups and a *P*<0.05 (two-tailed) was considered statistically significant.

## Results

### The combination of CpG and R848 enhances the protective efficacy of pVAX1-Sj26GST vaccination

**Figure 1:**
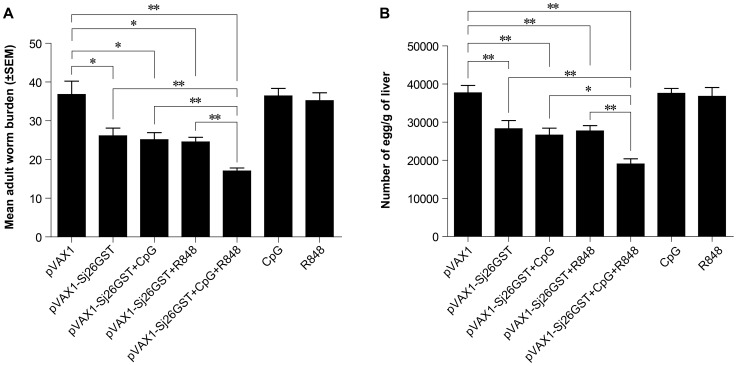
The combination of CpG and R848 increases protection induced by immunization with pVAX1-Sj26GST. C57BL/6 mice (6 mice per group) were injected with pVAX1-Sj26GST (50 µg), with or without 25 µg CpG, 25 µg R848, or both CpG and R848 (CpG+R848). Seven days after the last dose of vaccine, mice were sacrificed for the characterization of cellular and humoral immune responses. Alternately, 2 weeks after the final vaccination, mice from each group (8 per group) were challenged percutaneously with 40±1 *S. japonicum* cercariae. Six weeks later, the mice were sacrificed and perfused to determine worm burdens and liver egg burdens. The adult worm (A) and liver egg burdens (B) per mouse in each group were determined. The data are expressed as the mean ± SEM (n = 8) and are representative of 2 independent experiments. * *P*<0.05; ** *P*<0.01.

TLR ligands are potent inducers of immune activation and have been shown to augment vaccine efficacy. To assess the effect of TLR ligands on the protective efficacy of the pVAX1-Sj26GST vaccine, C57BL/6 mice were immunized 3 times at 2-week intervals with pVAX1, CpG, R848, pVAX1-Sj26GST, or pVAX1-Sj26GST plus CpG and/or R848. The degree of protection induced by vaccination was measured by the reduction in adult worm and egg burden. As shown in Figure 1, compared with the pVAX1-inoculated control group, mice inoculated with pVAX1-Sj26GST experienced a 29.04% decrease in worm burden and a 25.28% decrease in eggs in the liver (*P*<0.05) (Figure 1; Table 1). Meanwhile, addition of CpG or R848 during pVAX1-Sj26GST vaccination led to a 31.82% and 33.33% decrease in worms and a 29.75% and 26.83% decrease in eggs in the liver, respectively (Figure 1; Table 1). However, the combination of CpG and R848 during pVAX1-Sj26GST vaccination resulted in a higher decrease in worm burden (53.69%) and liver eggs (49.65%) compared with pVAX1-Sj26GST vaccination alone or with a single ligand, whereas CpG or R848 alone provided almost no reduction in worm burden or liver egg burden (Figure 1; Table 1). These results suggest that the combination of CpG and R848 significantly improves the protective efficacy of pVAX1-Sj26GST vaccination.

**Table 1:** The anti-worm and anti-egg effects in vaccinated-mice.

Group	worm burden (mean±SEM)	number of eggs/g liver (mean±SEM)	Percent reduction in worm burden	Percent reduction in egg production
**pVAX1**	36.75±3.48	37851.60±1943.62	/	/
**pVAX1-Sj26GST**	26.08±1.99	28282.70±2139.17	29.04	25.28
**pVAX1-Sj26GST+CpG**	25.06±1.85	26589.54±1826.65	31.82	29.75
**pVAX1-Sj26GST+R848**	24.5±1.24	27697.58±1411.21	33.33	26.83
**pVAX1-Sj26GST+CpG+R848**	17.02±0.80	19058.03±1377.80	53.69	49.65
**CpG**	36.4±1.95	37576.69±1280.64	9.52	7.26
**R848**	35.2±2.03	36760.76±2323.49	4.22	2.88

The mean worm/liver egg burdens were calculated using results from sixteen mice from two independent experiments (n = 8). Percent protection in the two independent experiments was calculated by comparing their results with the results obtained from the pVAX1 group. Results are expressed as means± standard error of the mean.

### The combination of CpG and R848 enhances splenocyte proliferation and the production of IgG and IgG2a antibody after vaccination with pVAX1-Sj26GST

**Figure 2:**
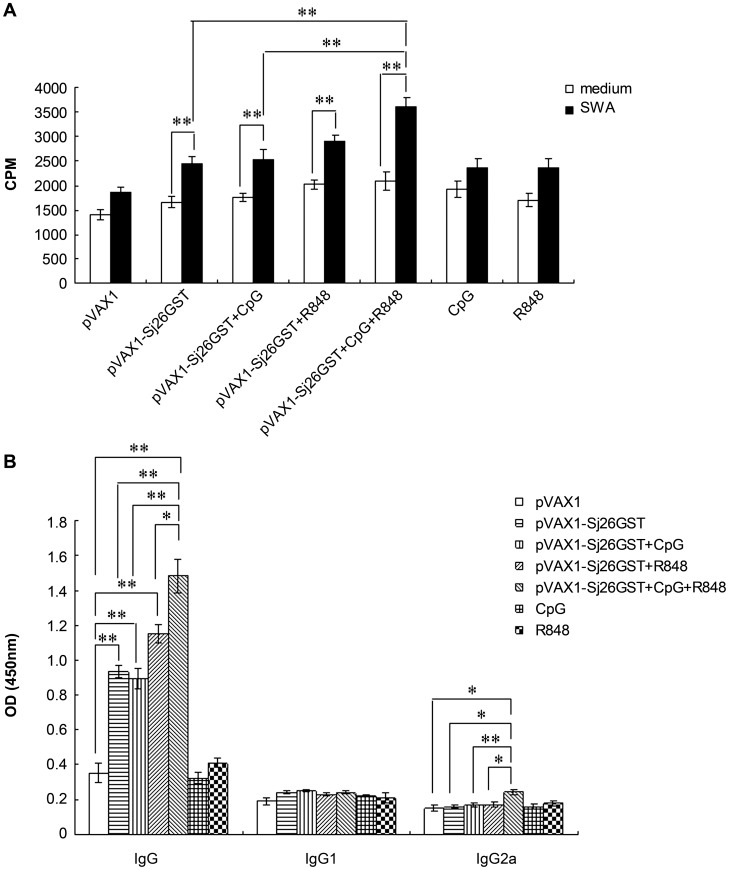
The combination of CpG and R848 enhances splenocyte proliferation and antibody production in pVAX1-Sj26GST–vaccinated mice. (A) Seven days after the last immunization with pVAX1-Sj26GST alone or with CpG, R848, or both (CpG+R848), splenocytes were harvested and antigen-specific proliferation was measured. Splenocytes (2×10^5^/well) from each mouse were incubated in the presence of SWA (15 µg/mL) or control media for 3 days in a 200 µL volume in 96-well plates. [^3^H] thymidine (0.5 µCi) was added to each well 16 h before the end of the incubation period. Data are expressed as the mean ± SEM (n = 6 per group) and are representative of 3 independent experiments performed in triplicate wells. * *P*<0.05. (B) IgG, IgG1, and IgG2a responses in immunized mice. Antibody responses to SWA (15 µg/mL) were determined by ELISA. Data are expressed as the mean ± SEM (n = 6 per group) of 18 mice from 3 independent experiments performed in triplicate wells. * *P*<0.05; ** *P*<0.01.

The results described above demonstrated that the combination of CpG and R848 improved the protection of pVAX1-Sj26GST vaccination. Thus, we investigated whether adjuvant CpG and R848 allows a more robust induction of immune responses after pVAX1-Sj26GST vaccination. To determine how these TLR ligands influence the immune response following schistosome antigen-specific stimulation, splenocyte proliferation and antibody production were assessed. Splenocytes were isolated from mice vaccinated with pVAX1, CpG, R848, pVAX1-Sj26GST, or pVAX1-Sj26GST plus CpG and/or R848 and stimulated with soluble worm antigen (SWA). To exclude the possibility that CpG and/or R848 induced splenomegaly by increasing splenocyte proliferation, mice were subcutaneously injected with CpG and/or R848 three times at 14-day intervals, after which the spleens were weighed and spleen cells counted. Injection of CpG and/or R848 did not induce splenomegaly (Figure S1), as the spleen weight and cell number from CpG and/or R848-injected mice were not significantly different that those of PBS-injected mice. As shown in Figure 2A, *in vitro* SWA stimulation significantly increased the proliferation of splenocytes isolated from pVAX1-Sj26GST-vaccinated mice. However, vaccination in combination with CpG and R848 resulted in higher splenocyte proliferation than vaccination with pVAX1-Sj26GST alone or together with CpG, and CpG or R848 alone led to almost no improvement in splenocyte proliferation. These results suggest that the combination of CpG and R848 enhanced antigen-specific T-cell proliferation during pVAX1-Sj26GST immunization.

To examine whether adjuvant CpG and R848 influences antibody production, the levels of specific SWA antibodies in the serum of vaccinated mice were examined. As shown in Figure 2B, pVAX1-Sj26GST vaccination causes a significant increase in antigen-specific IgG levels (*P*<0.01) compared with pVAX1 control inoculation (Figure 2B). However, vaccination in combination with CpG and R848 increased IgG levels more than vaccination with pVAX1-Sj26GST alone or with single ligands, and CpG or R848 alone provided almost no improvement in IgG levels. Furthermore, the combination of CpG and R848 induced a small but statistically significant increase in IgG2a level compared with pVAX1-Sj26GST alone or single ligands. No IgG1 response was observed in immunized mice, regardless of whether TLR ligands were used (Figure 2B). The end-point antibody titers are shown in Table 2. High levels of SWA-specific antibody titers were obtained from the total IgG from mice immunized with pVAX1-Sj26GST or pVAX1-Sj26GST plus CpG and/or R848. A robust antibody titer for IgM was also observed in the sera of the aforementioned vaccinated mice. However, the highest titers of IgG and IgM were observed in the group in which pVAX1-Sj26GST was combined with CpG and R848. No detectable levels of SWA-specific antibodies (IgG and IgM) were detected in mice immunized with pVAX1, CpG, or R848 alone. Taken together, these results indicate that the combination of CpG and R848 specifically enhances both splenocyte proliferation and IgG and IgG2a production during pVAX1-Sj26GST vaccination.

**Table 2:** Antibody titers in sera from vaccinated-mice.

Group	IgG	IgM
**pVAX1**	100±1.84	100±4.62
**pVAX1-Sj26GST**	1000±57.82	800±27.61
**pVAX1-Sj26GST+CpG**	1200±44.36	1200±82.17
**pVAX1-Sj26GST+R848**	3200±72.45	1600±79.59
**pVAX1-Sj26GST+CpG+R848**	4800±157.16	3200±94.63
**CpG**	100±5.14	100±3.12
**R848**	100±4.72	100±2.26

ELISA was performed with a pool of sera obtained by mixing equal volumes of serum collected from each mouse in a respective group. The values represent the mean of three replicates ± standard error.

### The combination of CpG and R848 augments the production of proinflammatory cytokines in pVAX1-Sj26GST–vaccinated mice

**Figure 3:**
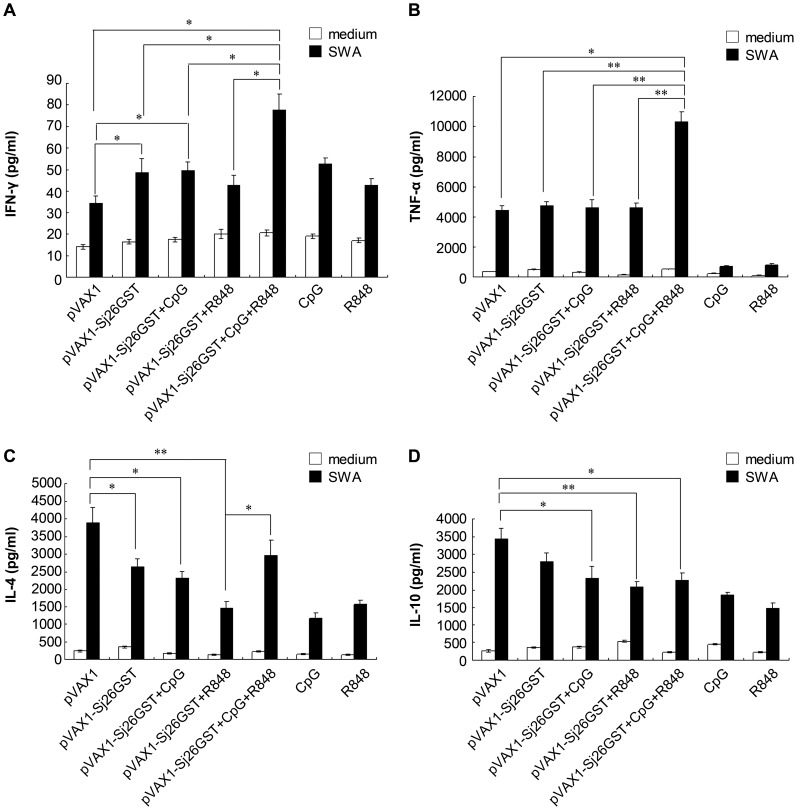
The combination of CpG and R848 upregulates proinflammatory cytokines in pVAX1-Sj26GST–vaccinated mice. Seven days after the last immunization with pVAX1-Sj26GST, alone or with CpG, R848, or both (CpG+R848), splenocytes (2×10^5^/well) from each mouse were incubated in in the presence of SWA (15 µg/mL) or control media for 3 days in a 200-µL volume in 96-well plates. Supernatants were collected after 72 h and tested for IFN-γ (A), TNF-α (B), IL-4 (C), or IL-10 (D). Bars represent the mean ± SEM (n = 6 per group) of 18 mice from 3 independent experiments performed in triplicate wells. * *P*<0.05; ** *P*<0.01.

To further investigate the influence of TLR ligands on the immune response, the levels of cytokines in splenocytes isolated from mice vaccinated with pVAX1, CpG, R848, pVAX1-Sj26GST, or pVAX1-Sj26GST plus CpG and/or R848 after SWA stimulation were examined. Compared with the pVAX1 control, pVAX1-Sj26GST vaccination significantly increased the production of IFN-γ (Figure 3A), whereas IL-4 levels decreased and TNF-α and IL-10 levels were not significantly changed (Figure 3B, 3C, and 3D). However, vaccination along with the combination of CpG and R848 resulted in higher IFN-γ and TNF-α levels than vaccination with pVAX1-Sj26GST alone or with single ligands (Figure 3A and 3B). Compared with pVAX1-Sj26GST plus R848, IL-4 levels were significantly elevated (P = 0.016) while IL-10 levels were slightly increased without statistical significance (P = 0.423) in mice vaccinated with pVAX1-Sj26GST plus CpG and R848 (Figure 3C and 3D). However, IL-4 and IL-10 levels in splenocytes from mice vaccinated with pVAX1-Sj26GST plus CpG and R848 were lower than in splenocytes from mice vaccinated with pVAX1 control (Figure 3C and 3D). Overall, these results demonstrate that the combination of CpG and R848 during pVAX1-Sj26GST vaccination causes the upregulation of proinflammatory cytokines.

### The combination of CpG and R848 reduces the population of CD4^+^CD25^+^ Tregs in pVAX1-Sj26GST–vaccinated mice

**Figure 4:**
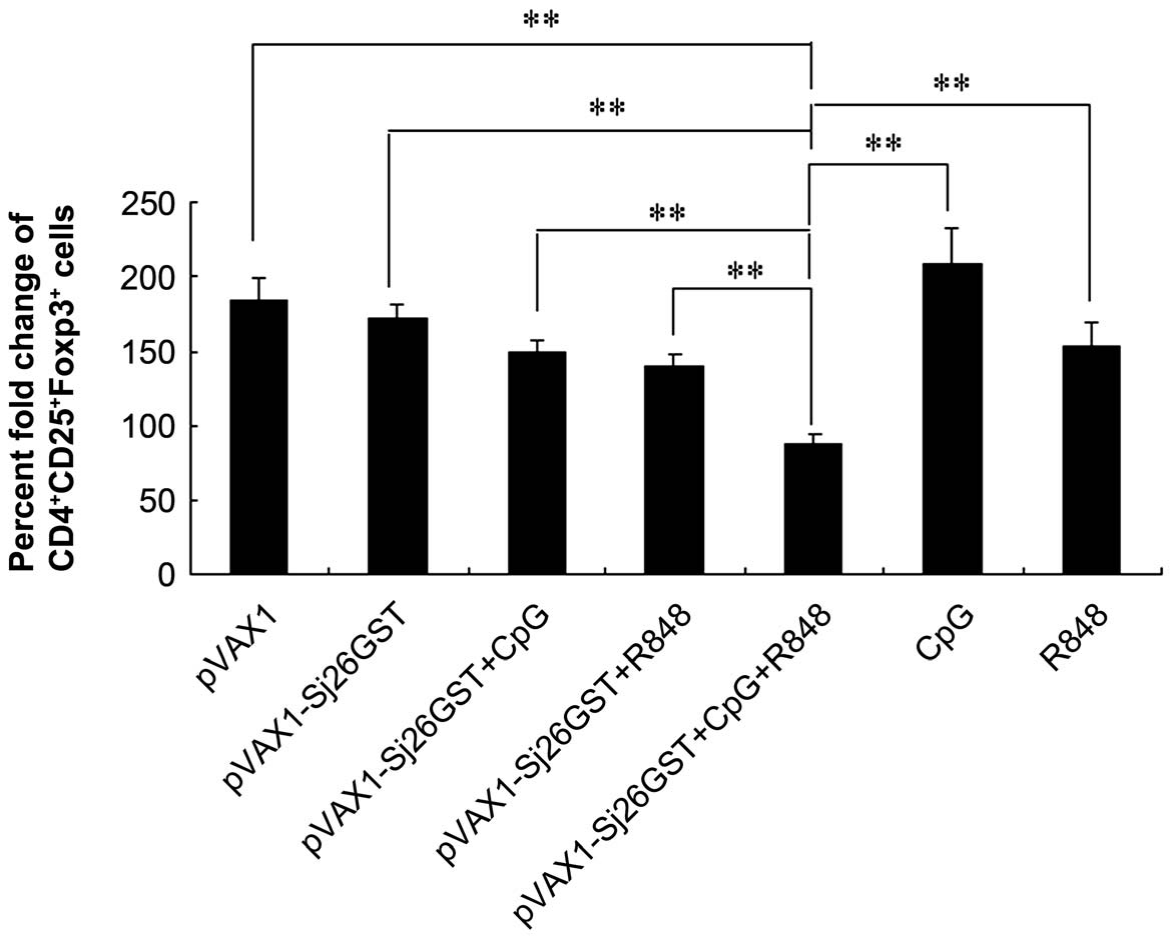
The combination of CpG and R848 inhibits the induction of CD4+CD25+ Tregs in pVAX1-Sj26GST–vaccinated mice. Seven days after the last immunization with pVAX1-Sj26GST, alone or with CpG, R848, or both (CpG+R848), splenocytes from each mouse were analyzed by flow cytometry analysis for CD3, CD4, CD25, and Foxp3 expression. The mean fold change of the percentage of CD4^+^CD25^+^Foxp3^+^ Tregs in mice at week 0 compared with 5 weeks after immunization with the indicated formulations is shown. Groups consisted of 6 mice at each time point. The results are expressed as the mean ± SEM of 12 mice from 2 independent experiments. ** *P*<0.01.

It has been reported that, while stimulating antigen-specific effector T cells, Tregs may be expanded to regulate effector T cells [32], [33]. Furthermore, our previous study demonstrated that pVAX1-Sj26GST immunization induces a significant increase of CD4^+^CD25^+^Foxp3^+^ Tregs that may be involved in the limited protection the vaccine confers [25]. We examined whether the TLR ligands enhanced the protection of pVAX1-Sj26GST by decreasing the number of Tregs during vaccination. To determine the impact of pVAX1-Sj26GST immunization with adjuvant CpG and R848 on CD4^+^CD25^+^ Treg frequency, splenocytes isolated from vaccinated mice were stained pre- and postimmunization for the Treg markers CD4, CD25, and Foxp3. As shown in Figure 4, in addition to the pVAX1-Sj26GST plus CpG and R848 group, other vaccinated mice showed an increase in CD4^+^CD25^+^ Tregs as judged by the fold change of Treg proportions after vaccination. Consistent with the results we described previously [25], both pVAX1- and pVAX1-Sj26GST–immunized mice induced an increase in the percentage of CD4^+^CD25^+^ Tregs after vaccination; however, there was no difference in the percentage of Tregs between the pVAX1 and pVAX1-Sj26GST groups. Inclusion of CpG or R848 alone in the vaccination did not affect Treg proportion; however, the combination of CpG and R848 significantly decreased the number of Tregs in immunized mice compared with mice vaccinated with pVAX1-Sj26GST alone, pVAX1-Sj26GST together with single ligands, or the control groups. Single CpG or R848 also induced an increase in the CD4^+^CD25^+^ Treg population after vaccination (Figure 4). These results suggest that the combination of CpG and R848 might prevent the expansion of CD4^+^CD25^+^ Tregs and thereby improve the immune response and protection of pVAX1-Sj26GST vaccination.

### The combination of CpG and R848 inhibits CD4^+^CD25^+^ Treg immunosuppression *in vitro*

**Figure 5:**
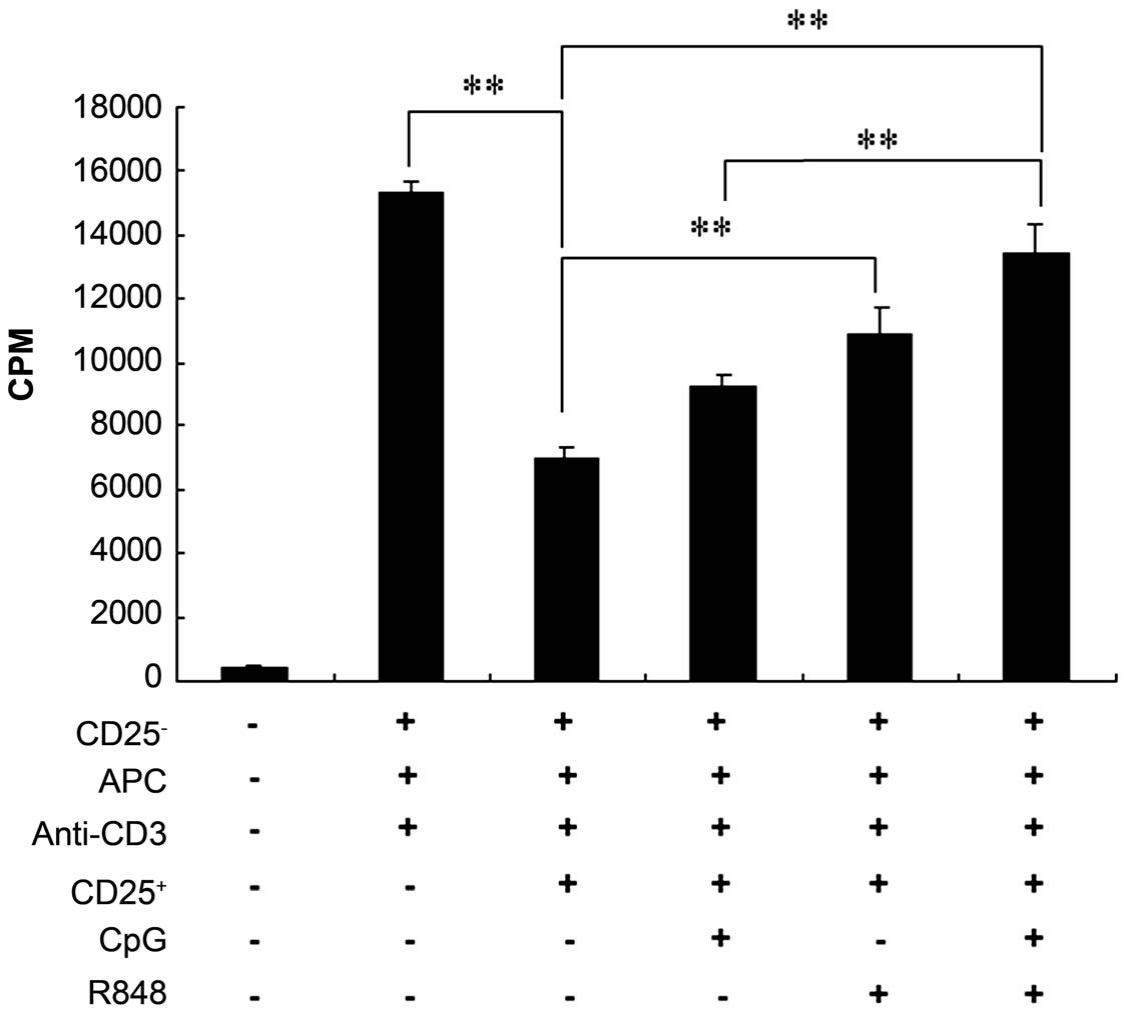
The combination of CpG and R848 inhibits CD4+CD25+ Tregs function *in vitro*. Isolated CD4^+^CD25^−^ T cells (1×10^5^/well) from naïve mice were cultured with or without CD4^+^CD25^+^ Tregs (5×10^4^/well), irradiated APC (1×10^5^/well) and 1 µg/mL anti-CD3 in 96-well plates in the presence or absence of 3 µg/mL CpG, R848, or both for 72 h. Proliferation was determined by measuring ^3^H-thymidine incorporation for the last 16 h of the experiment. Data are expressed as the mean ± SEM of 3 experiments performed in triplicate. ** *P*<0.01.

TLR8 and TLR9 ligands have been shown to directly impair Treg function in humans or rats [20], [21]. In order to examine further the effects of CpG and R848 on Treg activity in our system, CD4^+^CD25^−^ T cells (responder cells) were sorted and cocultured with CD4^+^CD25^+^ T cells from naïve mice. Figure 5 shows that following stimulation with anti-CD3 antibody, CD4^+^CD25^+^ T cells were highly effective at suppressing CD4^+^CD25^−^ T-cell proliferation. Conversely, adding CpG moderately reduced the inhibition of T-cell proliferation. However, adding either R848 or both CpG and R848 significantly inhibited Treg-mediated suppression of T-cell proliferation. Compared to the combination of CpG and R848, CpG alone induced lower level in inhibiting T-cell proliferation, whereas R848 alone showed no statistical significant reduction in Treg-mediated inhibition (Figure 5). These results suggest that the combination of CpG and R848 not only reduces the Treg population in vaccinated mice *in vivo* (Figure 4) but also inhibits Treg function *in vitro*.

### R848 or its combination with CpG induced higher levels of proinflammatory cytokines in a conventional *in vitro* suppression assay

**Figure 6:**
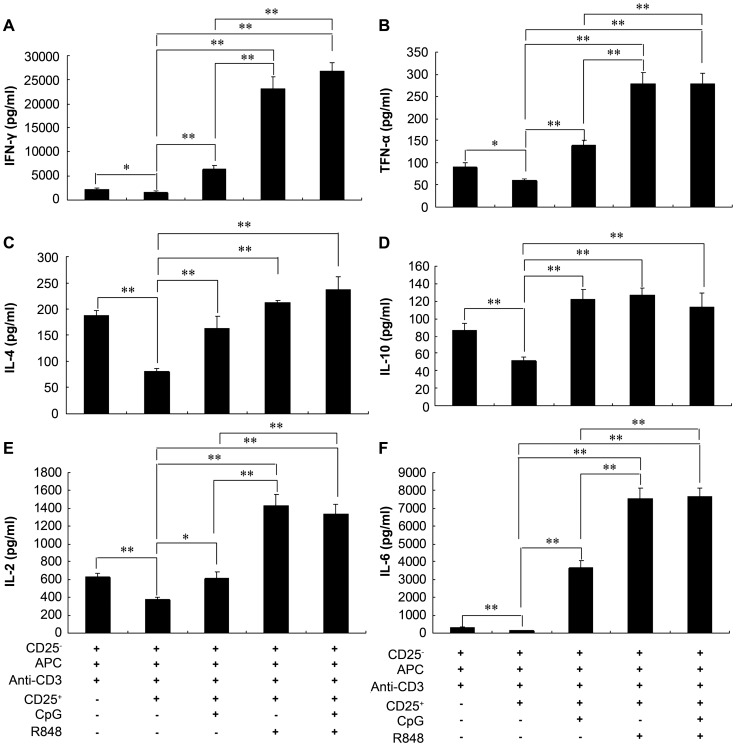
R848 or its combination with CpG induces a panel of proinflammatory cytokines in a conventional *in vitro* suppression assay. Isolated CD4^+^CD25^−^ T cells (1×10^5^/well) from naïve mice were cultured with or without CD4^+^CD25^+^ Tregs (5×10^4^/well), irradiated APC (1×10^5^/well) and 1 µg/mL anti-CD3 in 96-well plates in the presence or absence of 3 µg/mL CpG, R848, or both. Supernatants were collected after 72 h and tested for IFN-γ (A), TNF-α (B), IL-4 (C), IL-10 (D), IL-2 (E), or IL-6 (F). Data are expressed as the mean ± SEM from 2 experiments performed in triplicate wells. * *P*<0.05; ** *P*<0.01.

It has been reported that cytokines secreted by APCs in response to TLR ligands are important to counteract the immunosuppressive effects of CD4^+^CD25^+^ Tregs [34], [35]. Several reports have also shown that a variety of proinflammatory cytokines can overcome Treg suppression during infection or in an inflamed environment, including IL-2 [36], IL-4 [37], IL-6 [21], [38], and TNF-α [39], [40]. To investigate the impact of CpG and R848 on cytokine production *in vitro*, CD4^+^CD25^−^ cells from naïve mice were cocultured with naïve murine CD4^+^CD25^+^ cells, irradiated APCs, and anti-CD3 in the presence or absence of CpG, R848, or both and cytokine production in the supernatants was evaluated. As shown in Figure 6, consistent with previously reported observations that CD4^+^CD25^+^ Tregs suppress the production of IFN-γ, TNF-α, IL-4, IL-10, IL-2, and IL-6 [41], [42], CpG or R848 significantly enhanced the production of these cytokines. Compared to the combination of CpG and R848, CpG alone induced lower levels of IFN-γ, TNF-α, IL-2, and IL-6, whereas R848 alone induced almost equal levels of the above-mentioned cytokines (Figure 6). Compared to IL-10 levels, IFN-γ, TNF-α, IL-4, IL-2, and IL-6 levels were remarkably high in a conventional *in vitro* suppression assay after adding CpG and/or R848 (Figure 6). The elevated levels of proinflammatory cytokines in the presence of CpG and/or R848 correlates with inhibition of Treg function as described in Figure 5. Thus, these results suggest that R848 or its combination with CpG induces higher levels of proinflammatory cytokines, which may help break the immunosuppression of CD4^+^CD25^+^ Tregs in a conventional *in vitro* suppression assay.

### The combination of CpG and R848 reduces the induction of Foxp3-expressing T cells *in vitro*

**Figure 7:**
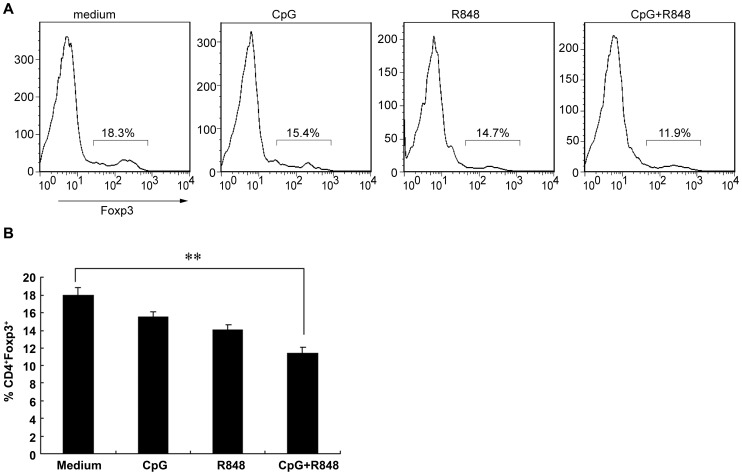
The combination of CpG and R848 reduces the induction of Foxp3-expressing T cells *in vitro*. Splenocytes from naïve mice were stimulated *in vitro* with 3 µg/mL CpG, R848, or both for 48 h and then subjected to flow cytometry analysis for CD3, CD4, CD25, and Foxp3 expression. (A) Representative flow cytometry data indicating the percentages of Foxp3 expression in CD4^+^ T cells. (B) The percentage of CD4^+^Foxp3^+^ T cells. Data are expressed as the mean ± SEM of 12 mice from 2 independent experiments. ** *P*<0.01.

It has been reported that the transcription factor Foxp3 is required for the suppressive activity of Tregs, and its expression in non-regulatory cells converts them into immunosuppressive cells [43]. Furthermore, it has been reported that IFN-γ [44], IL-4 [44], IL-6 [45], and TNF-α [40] can inhibit Foxp3 expression. The elevated amounts of proinflammatory cytokines conferred by the combination of CpG and R848 in a conventional *in vitro* suppression assay may inhibit the expression of Foxp3 and further affect Treg function and conversion. To test whether the inhibition of Treg function by CpG and R848 was related to the reduction of Foxp3 expression, splenocytes from naïve mice were isolated and exposed to CpG and/or R848 for 48 h *in vitro*. Foxp3 expression was then analyzed by flow cytometry (FCM). FCM showed that the population of Foxp3-expressing splenocytes was significantly reduced in the presence of CpG and R848. Single CpG or R848 induced a reduction in Foxp3 expression compared with medium alone that was not statistically significant (Figure 7). These results are consistent with the findings that the combination of CpG and R848 decreased the population of CD4^+^CD25^+^ Tregs in pVAX1-Sj26GST–vaccinated mice and inhibited Treg function *in vitro*.

## Discussion

TLR ligands stimulate innate, adaptive, and regulatory immune responses and as vaccine adjuvants represent a promising approach to stimulating strong immune responses and enhancing vaccine-induced protection [46]. Engagement of TLR9 by CpG enhances immune responses to co-delivered antigens in animal models and are now being developed for clinical use as either vaccine adjuvants or immune therapeutics by Coley Pharmaceuticals (Pfizer) and Dynavax Technologies, among others [46]. The TLR7/8 ligand R848 has been approved by the U.S. Food and Drug Administration for use as a stand-alone entity [47], and was proven to enhance the immune response to co-administered antigens as a vaccine adjuvant [48], [49]. However, the impact of CpG and R848 on vaccines against schistosomiasis, a disease that poses a significant public health concern in many tropical countries, is unknown, and was the subject of this investigation.

In the present study, we demonstrated that immunization with pVAX1-Sj26GST combined with CpG and R848 as adjuvants induces a stronger protection compared with pVAX1-Sj26GST alone or with either single ligand. It has been reported that the use of TLR ligands as adjuvants can elicit more vigorous immune responses against infection and cancer [46], [50]. Indeed, immunization with pVAX1-Sj26GST combined with CpG and R848 resulted in a significant increase in vaccine-induced splenocyte proliferation and IgG and IgG2a levels. These results are consistent with another study demonstrating that CpG and R848 are the strong Th1-biased adjuvants [51], because Th1-associated IgG2a was significantly increase in the group in which pVAX1-Sj26GST was combined with CpG and R848. However, CpG or R848 alone did not enhance protection and immune responses conferred by the vaccine. This finding is consistent with a recent publication showing that TLR ligands as combination adjuvants induce qualitative changes in T-cell responses needed for antiviral or antiparasite protection in mice [52], [53]. Furthermore, consistent with the current work, Ahmad and colleagues reported that use of R848 as an immunopotentiating agent slightly boosted the protective effects of Sm-p80, now considered a leading putative vaccine candidate antigen from *Schistosoma mansoni* (*S.mansoni*), in both the “DNA prime-protein boost” and “recombinant protein” immunization approaches in mice [24].

The quantification of cytokines in splenocyte culture supernatants indicated that pVAX1-Sj26GST vaccination induces significantly increased IFN-γ levels and decreased IL-4 levels compared with vaccinated control pVAX1-treated mice. However, combining CpG and R848 with pVAX1-Sj26GST augmented the production of IFN-γ and TNF-α in vaccinated mice. Elevation of IFN-γ and TNF-α in response to the combination of CpG and R848 may contribute to the enhanced protection conferred by pVAX1-Sj26GST vaccination. Because the protection induced by many schistosoma vaccines was associated with elevated production of IFN-γ and TNF-α [54], [55] , our data also suggest that the activation of more than one TLR could be an effective approach to optimize immune responses in vaccination. Consistent with these findings, Lanzavecchia and colleagues [56] reported that synergistic TLR stimulation mimics pathogens that contain several TLR ligands and induces enhanced and sustained T helper type 1 responses in DCs. Furthermore, several studies have shown that certain TLRs enhance T cell-mediated immune responses through synergistic activation of DCs when their ligands are detected in pairs [56], [57] or through induction of high levels of proinflammatory cytokines by simultaneously activating different signaling pathways [34], [52]. The enhancement of T-cell responses and proinflammatory cytokine secretion may therefore improve the protection conferred by pVAX1-Sj26GST vaccination.

Apart from enhancing effector T-cell proliferation and cytokine production, TLR ligands could be involved in the modulation of adaptive immunity, including Treg-mediated immune suppression in vaccination [58], [59]. Furthermore, our previous study demonstrated that induction of CD4^+^CD25^+^ Tregs after pVAX1-Sj26GST vaccination may explain the limited protection conferred by this vaccine [25]. We thus hypothesized that enhancement of immune responses and protection conferred by the combination of CpG and R848 may be related to the inhibition of Treg induction after pVAX1-Sj26GST vaccination. Indeed, we did see a small but significant decrease in CD4^+^CD25^+^ Tregs after vaccination with pVAX1-Sj26GST plus CpG and R848. However, use of CpG or R848 alone only slightly affected the Treg population, which is consistent with other viral studies suggesting that combination of TLR ligands prevents expansion of Foxp3^+^ Tregs and thereby improves T cell responses [53]. However, Hoelzinger and colleagues report that intratumoral delivery of CpG ODN strongly reduces the levels of Tregs within the tumor [60]. Hackl and coworkers demonstrated that TLR7 ligands, e.g., R848, reduce the number of Tregs generated *de novo* from naïve murine T cells *in vitro* and *in vivo* [45]. Whether these differences are due to different host systems, different disease models, or different vaccine formulations remains to be investigated in future studies. The decreased population of CD4^+^CD25^+^ Tregs in response to the combination of CpG and R848 may therefore improve immune responses and protection in pVAX1-Sj26GST-vaccinated mice.

There is evidence that TLR signaling can modulate the suppressive functions of Tregs [35], [39]. Based on these studies, we investigated the effects of CpG and R848 on Treg-mediated suppression *in vitro*. In contrast to the human TLR8 ligands CpG-A and poly (G10) [21] and the TLR9 ligand CpG ODN [20], which abrogate or reverse the immunosuppressive function of CD4^+^CD25^+^ Tregs, we found that CpG did not inhibit Treg suppression, whereas R848 alone or in combination with CpG significantly inhibited the function of Tregs. This finding support the notion that, despite several structural and functional properties shared by all the members of the TLR family, the signaling through various TLRs elicits qualitatively and quantitatively diverse immune responses [61], such as their impact on Treg function.

It has been suggested that exposure to inflammatory cytokines released by APCs can render Tregs defective in immunosuppression [41]. For example, IL-6 production by TLR-activated DCs can inhibit the suppressive function of Treg cells [38]. Furthermore, exposure to TNF-α can inhibit the function of Tregs by signaling through TNF receptor II [40]. Consistent with a previous study reporting that CD4^+^CD25^+^ Tregs are able to suppress T-cell proliferation and cytokine production [62], our study demonstrated that the presence of CpG and/or R848 in a conventional *in vitro* suppression assay induces a panel of inflammatory cytokines, including IFN-γ, TNF-α, IL-4, IL-10, IL-2, and IL-6, that may inhibit Treg suppression. Although IL-10 is a major anti-inflammatory cytokine induced by TLR signaling and functions to inhibit production of TLR-induced proinflammatory mediators, such as TNF [63], this study shows that elevated levels of IL-10 in the presence of CpG and/or R848 in an *in vitro* suppression assay is insufficient to overcome the strong inflammatory context caused by other cytokines. Furthermore, CpG and R848 reduce the expression of Foxp3 in CD4^+^ T cells *in vitro*, which is indispensable in Treg development and function [44], [64]. Consistent with the above-mentioned cytokine production observed in an *in vitro* suppression assay, a variety of cytokines has been reported to inhibit Treg function by inhibiting Foxp3 expression, including IFN-γ [44], TNF-α [40], IL-4 [44], and IL-6 [45]. Although it remains to be determined whether the increased production of inflammatory cytokines induced by CpG and R848 directly stimulates CD4^+^CD25^−^ effector T cells or indirectly acts on APCs, these results strongly suggest that the combination of CpG and R848 contributes to the activation and expansion of effector T cells, increases cytokine secretion, and interferes with Treg function by downregulating Foxp3 expression. Although the *in vitro* assays of TLR ligands on Tregs fail to completely mimic the *in vivo* milieu, they lead us to speculate that the downregulation of Foxp3 expression not only affects Treg function *in vitro*, but also may impair the generation of Tregs after vaccination *in vivo*, thereby reducing the number of CD4^+^CD25^+^ Tregs in mice vaccinated with pVAX1-Sj26GST together with CpG and R848. These results are consistent with a previous study demonstrating that activation of DCs by TLR7 ligands leads to downregulation of Foxp3 expression after initial induction and consequently lowers Treg numbers in DC–T-cell cocultures *in vitro* [45]. Furthermore, single TLR ligands less potently decrease CD4^+^Foxp3^+^ T cells, whereas combined TLR ligands might prevent Foxp3^+^ Treg expansion and thereby improve T-cell responses [53]. However, R848 induced higher cytokine production than CpG in a conventional *in vitro* suppression assay. This is in contrast to other studies on cytokine secretion by splenocytes stimulated with CpG or R848 in which CpG was a greater stimulant of IL-6 and IL-12 secretion than R848 and R848 was superior to CpG in promoting IL-10 secretion [65]. The differences between CpG and R848 in inducing cytokine production might be related to the differences in their respective TLR signaling pathways, differences in the stability of CpG and R848 interactions with the ligands, and/or differences in the stability of the molecules in cells in the conventional *in vitro* suppression assay. Although there is no direct evidence that R848 is superior to CpG in induction of inflammatory cytokines, Martín-Fontecha and colleagues reported that R848, but not CpG1826, can recruit NK cells to produce IFN-γ and prime T cells for the induction of TH1 cells [29]. Further analysis is needed to determine why R848 induced more cytokines than CpG and which cells produced these cytokines in a conventional *in vitro* suppression assay. A greater understanding of the cellular events triggered by single or combinations of TLRs will be valuable in the rational design of more successful TLR-based immunotherapies and vaccination strategies.

However, It should be noted that although cooperation among TLRs during infection and vaccination may result in more robust immune responses and protection, if not properly controlled, these strong responses can result in immunopathologies such as autoimmunity [66]. Indeed, the use of the TLR7 and TLR8 agonist imiquimod in patients with cancer exacerbates psoriasis [67]. Thus, TLR-regulated Treg activity and conversion could enhance pathogen clearance but also increase the risk of autoimmune reactions. Future studies using a TLR-based vaccine strategy are required to evaluate this possibility.

In conclusion, this work demonstrates that the combination of CpG and R848 increases the proliferation of splenocytes and IgG levels and improves disease protection after immunization with the *S. japonicum* vaccine pVAX1-Sj26GST. This enhancement of protection may be related to the inhibition of Treg expansion and function, as the combination of CpG and R848 may impair the Treg development and function by upregulating the secretion of proinflammatory cytokines and decreasing Foxp3 expression. In combination with the vaccine, TLR ligands may protect the effector response from Treg-mediated suppression, thereby eliciting the appropriate immune response to improve vaccine efficacy. Our findings support the notion that, similar to an infection, vaccination also may allow Tregs to expand concurrently with T cells [68]. However, the addition of paired TLR ligands as adjuvants induces an proinflammatory setting which acts either by direct inhibition of Treg suppression or rescue of Teffs from Treg-mediated suppression to allow expansion of antigen-specific T cells against *S. japonicum*. Therefore, modulation of Tregs by adjuvant TLR ligand combinations may represent an attractive strategy to enhance the efficacy of vaccination against pathogens.

## Supporting Information

Figure S1 **CpG and/or R848 did not induce splenomegaly in mice.** C57BL/6 mice (6 mice per group) were subcutaneously injected with PBS, 25 µg CpG, 25 µg R848, or both CpG and R848 (CpG+R848) three times at 14-day intervals, Seven days after the last injection, mice were sacrificed for the characterization of spleen weight (A) and cell numbers (B). Spleen weight is presented as spleen weight (mg)/total body weight (g). The data are expressed as the mean ± SEM (n = 6) and are representative of 2 independent experiments.
(TIF)
