# Supplementary figures and images for: Combined TLR7/8 and TLR9 Ligands Potentiate the Activity of a Schistosoma japonicum DNA Vaccine
Source: PLoS Negl Trop Dis. 2013 Apr 4;7(4):e2164. doi: 10.1371/journal.pntd.0002164 (PMC3617091; doi:10.1371/journal.pntd.0002164)

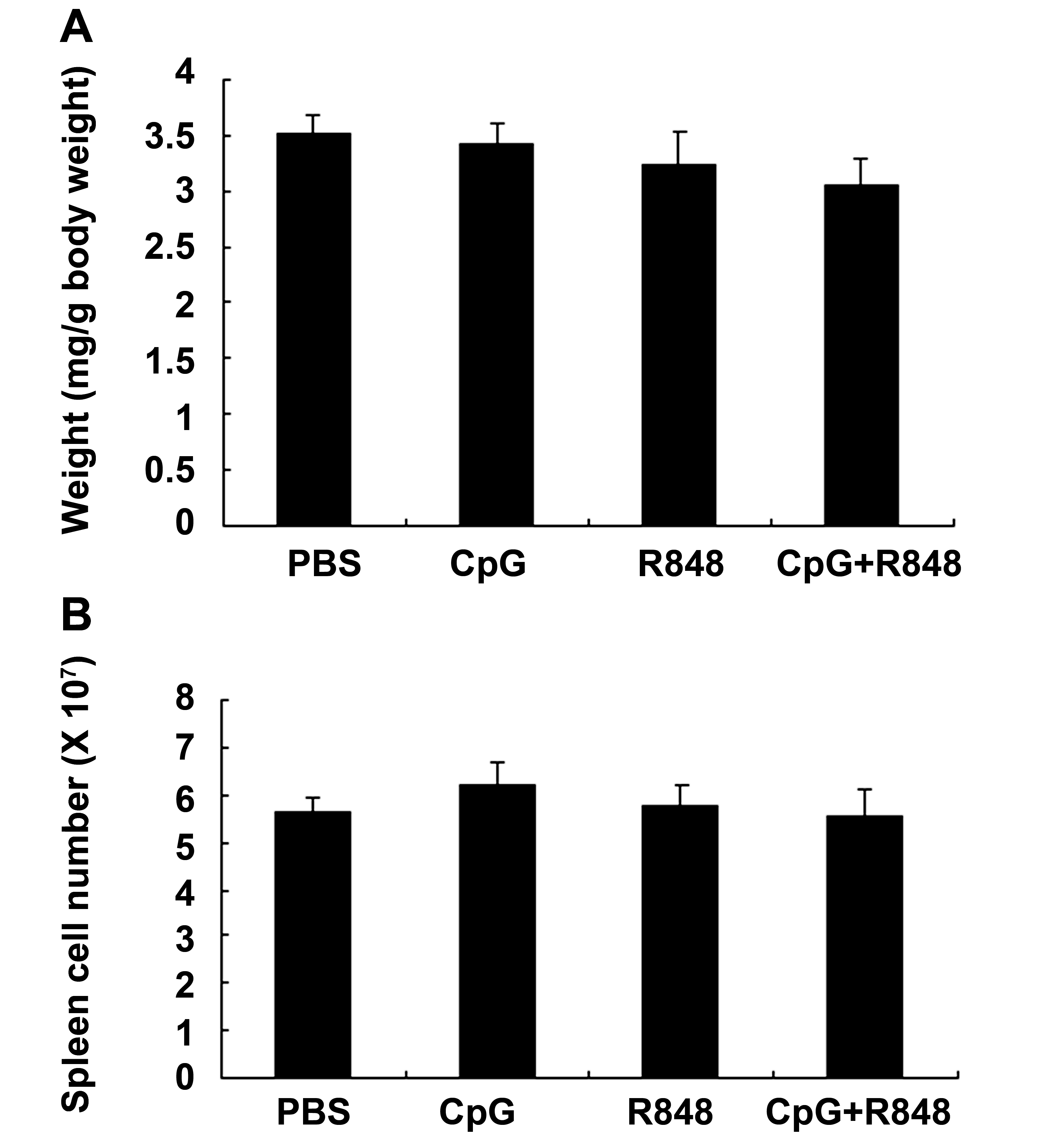

Supplement: Figure S1 — CpG and/or R848 did not induce splenomegaly in mice. C57BL/6 mice (6 mice per group) were subcutaneously injected with PBS, 25 µg CpG, 25 µg R848, or both CpG and R848 (CpG+R848) three times at 14-day intervals, Seven days after the last injection, mice were sacrificed for the characterization of spleen weight (A) and cell numbers (B). Spleen weight is presented as spleen weight (mg)/total body weight (g). The data are expressed as the mean ± SEM (n = 6) and are representative of 2 independent experiments. (TIF) [file pntd.0002164.s001.tif]
